# Psychotomimetic compensation versus sensitization

**DOI:** 10.1002/prp2.1217

**Published:** 2024-06-23

**Authors:** Ari Brouwer, Robin L. Carhart‐Harris, Charles L. Raison

**Affiliations:** ^1^ Department of Human Development and Family Studies, School of Human Ecology University of Wisconsin‐Madison Madison Wisconsin USA; ^2^ Department of Neurology and Psychiatry University of California San Francisco San Francisco California USA; ^3^ Department of Psychiatry, School of Medicine and Public Health University of Wisconsin‐Madison Madison Wisconsin USA; ^4^ Vail Health Behavioral Health Innovation Center Vail Colorado USA; ^5^ Center for the Study of Human Health Emory University Atlanta Georgia USA; ^6^ Department of Spiritual Health Emory University Woodruff Health Sciences Center Atlanta Georgia USA

**Keywords:** psychedelic, psychosis, pychotomimetic, schizophrenia, sensitization, stress, substance use

## Abstract

It is a paradox that psychotomimetic drugs can relieve symptoms that increase risk of and cooccur with psychosis, such as attention and motivational deficits (e.g., amphetamines), pain (e.g., cannabis) and symptoms of depression (e.g., psychedelics, dissociatives). We introduce the ideas of psychotomimetic compensation and psychotomimetic sensitization to explain this paradox. Psychotomimetic compensation refers to a short‐term stressor or drug‐induced compensation against stress that is facilitated by engagement of neurotransmitter/modulator systems (endocannabinoid, serotonergic, glutamatergic and dopaminergic) that mediate the effects of common psychotomimetic drugs. Psychotomimetic sensitization occurs after repeated exposure to stress and/or drugs and is evidenced by the gradual intensification and increase of psychotic‐like experiences over time. Theoretical and practical implications of this model are discussed.

## 1 INTRODUCTION

Well known psychotomimetic agents include classic psychedelics such as mescaline, psilocybin and its active metabolite psilocin, and LSD ^1^; cannabis and its psychoactive constituent Delta‐9‐Tetrahydrocannabinol (THC)^2^; dissociative anesthetics such as ketamine ^3^; and frequent high doses of dopamine‐enhancing drugs like methamphetamine.^4^ The capacity for these drugs to elicit psychotomimetic “psychosis mimicking” states^5^ is supported by preclinical studies and animal models of psychosis,^6^ psychological tests and subjective reports in humans,^7^, ^8^ as well as epidemiological data on substance use in populations who are at risk for and experience psychosis.^9^, ^10^

However, the fact that psychotomimetics are used to treat pain,^11^ attention deficits^12^ and depression^13^ raises questions about the nature of psychotic symptoms and whether they reflect compensatory (albeit dysfunctional) responses to stress or homeostatic imbalance. It has been argued before that psychosis is a mere indicator of homeostatic imbalance, illness, or enduring social dysfunction,^14^ but this view does not explain how temporary psychotic‐like states elicited by psychotomimetics, or induced intentionally by ritual or ascetic practices, can be therapeutic or at least lead to short‐term increases in well‐being.^15^, ^16^ Nor does it account for the fact that all rapid‐acting antidepressants (psychedelics, ketamine, sleep deprivation) are potent psychotomimetics.^13^ Or how, over time, an individual can become sensitized to a psychotomimetic stress response, in general or in response to specific cues (e.g., social threat), and thereby more prone to recurring and severe psychotic experiences.^17^, ^18^, ^19^, ^20^, ^21^ In all these cases we believe that the ideas of psychotomimetic compensation and sensitization provide a parsimonious and falsifiable explanation for these phenomena.

## 2 PSYCHOTOMIMETIC COMPENSATION

Arthur Koestler: You must admit that these drugs cause psychosis. A temporary psychosis… Would you say its therapeutic?

Timothy Leary: That's what the effect should be called. TTP. INSTANT MYSTICISM. Temporary Therapeutic Psychosis.^22^ pg. 152

**FIGURE 1:**
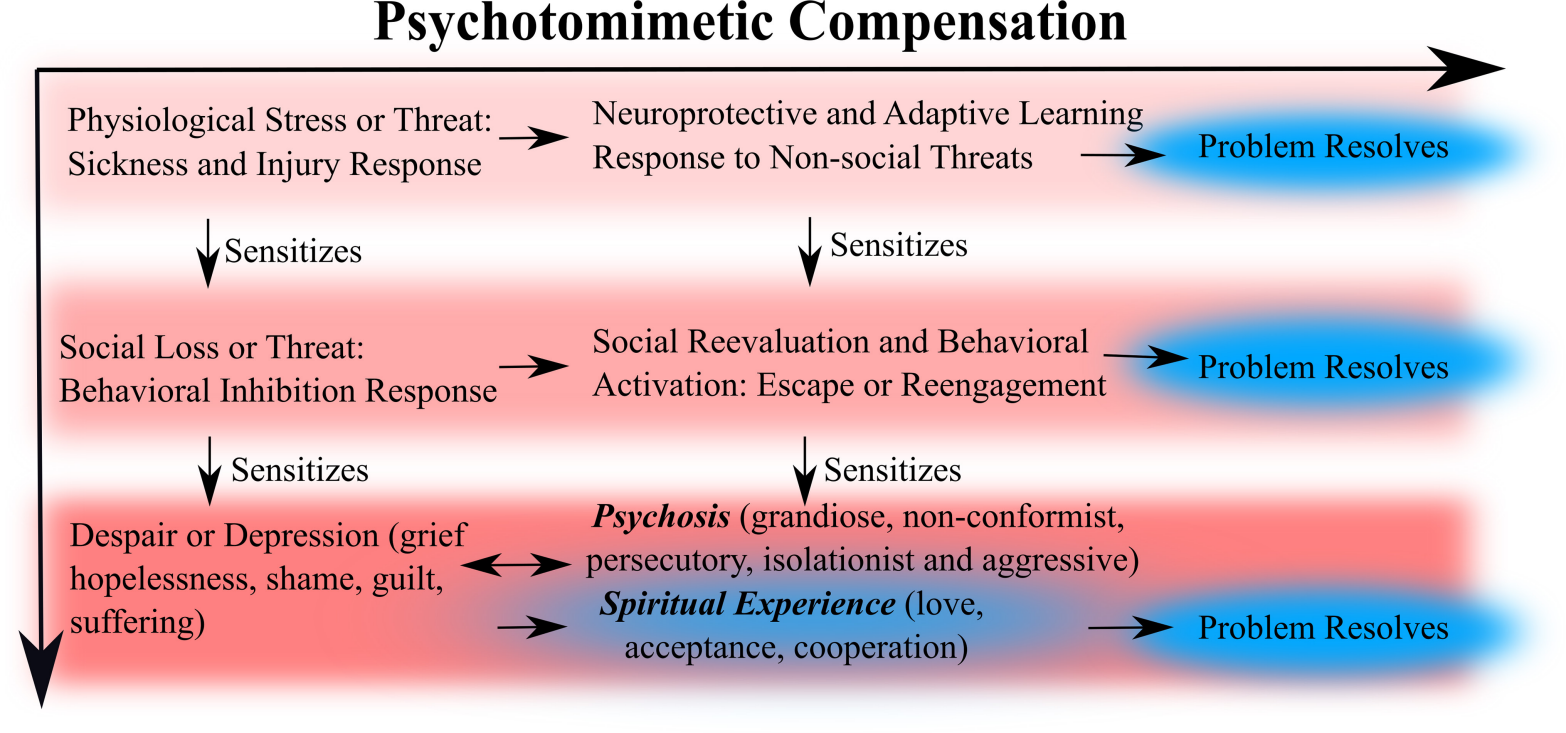
Similar feelings (e.g., exhaustion) and behaviors (e.g., immobility) are elicited by injury, sickness, social threat or loss, and likely sensitize individuals to future inhibitory responses to stress as well as depression. Psychotomimetic signaling compensates against these inflammatory and inhibitory responses by increasing neuroprotection, energy, motivation, behavioral reevaluation and reengagement, and is generally adaptive, helping individuals to move beyond or resolve the sources of their distress. Intense psychotomimetic compensation, however, due to intense or chronic stress, could facilitate psychotic or spiritual experiences, the boundaries of which overlap to some degree. However, as these experiences recur over time, at risk individuals may become sensitized and vulnerable to psychosis.

People use psychotomimetic drugs for various reasons, but one is to relieve symptoms of pain, anxiety and depression.^23^, ^24^ It follows that psychotomimetic drugs may “mimic” an endogenous process of psychotomimetic compensation that relieves subjective symptoms of fatigue, pain, anxiety and depression. Indeed, psychotic symptoms (e.g., hallucinations) occur more frequently in contexts of chronic or high stress—for example, during fever,^25^ sleep deprivation,^26^ starvation,^27^ low oxygen conditions^28^ or socially stressful scenarios that elicit fear or shame.^29^ Psychotomimetic compensation—mirroring the effects of psychotomimetic drugs—may help normalize metabolic function,^30^, ^31^ reduce inflammation,^32^ prevent brain damage^33^ and ameliorate signs and symptoms of injury and illness such as lethargy, behavioral inhibition, social withdrawal, pain, low motivation, sadness and shame (Figure 1).

It might not be immediately obvious that a compensatory response to starvation or hypoxia could also compensate against depression, but from an evolutionary perspective, parsimonious explanations are generally preferable. We think psychotomimetic responses to physiological threats and non‐social environmental dangers have been evolutionarily co‐opted to protect against psychosocial threats. Similar explanations have been applied to depression‐like socioemotional responses (e.g., shame, grief, guilt) that appear to co‐opt more basic physiological responses to sickness and injury, such as sadness and lethargy.^34^ Within this framework, endogenous psychotomimetic signaling not only protects against immediate threat but also compensates against the deleterious effects of enduring sickness and injury responses (e.g., inflammation), social loss and threat, and depression (Figure 1).

## 3 PHENOMENOLOGY OF PSYCHOTOMIMETIC COMPENSATION

In order to understand how psychotic symptoms reflect compensatory processes, we must first acknowledge that psychosis reflects an abnormal, extreme, recurring and dysfunctional experiential state. In contrast, a less intense or time‐limited process of psychotomimetic compensation can elicit pleasant experiences that may or may not exhibit mild psychotic‐like or dissociative‐like features depending upon the intensity of stressor and subsequent compensation. For example, many individuals use saunas, exercise, fast or deprive themselves of sleep to increase feelings of wellbeing, and all these practices have antidepressant effects.^35^, ^36^, ^37^, ^38^ The desired subjective effects of these practices often occur after an initial period of mild discomfort. In the case of exercise, an initial period of unpleasantness, pain, exhaustion, confusion, hunger or tiredness may give way to a “flow”,^39^ “runners high”^40^ or “second‐wind”^41^ type state in which one experiences the desired increase in body‐high, perceptual clarity, creativity, euphoria and energy.

As the duration and intensity of stress increase, the likelihood of florid psychotic and dissociative symptoms increases. One does not run for a minute and achieve a runner's high, and a mild runner's high is not likely to elicit the misperceptions and hallucinations that are more common during ultra‐long distance running.^42^, ^43^ Likewise, the psychotomimetic effects of sleep deprivation become more psychotic‐like over time, evolving from distortions and hallucinations in the visual domain to include auditory hallucinations and delusional ideation.^26^ The idea that psychotic‐like experiences reflect compensatory processes, and not just dysfunction, is further supported by the fact that the mere threat of physical harm can elicit psychotomimetic compensation. Experiences of time‐expansion during car crashes^44^ and dissociation in the face of other dangers are two examples of this phenomenon.

In the clinical arena, psychotomimetic compensation manifests as a shift away from depressogenic emotions and behaviors towards psychotogenic emotions and behaviors, which helps to explain both the causal relationships and phenomenological distinctions between depression and florid psychosis. Transient depression‐like states can be adaptive, serving as brakes to stop behavioral perseverance in the face of negative feedback such as criticism, social loss, or failure.^45^

Psychotomimetic compensation helps resolve depression‐like states by promoting reevaluation and reengagement (see Figure 1). This compensatory process appears pathogenically amplified in bipolar mood and psychotic disorders. For example, a sense of connection and meaning is typically lost in depression^46^ or lacking in premorbid stages of schizophrenia,^47^ while it is amplified in mania^48^ and first‐episode psychosis.^47^ Whereas depression is characterized by negative self‐evaluations^49^ and reduced self‐serving bias,^50^ delusional ideation and psychosis are associated with externalizing and self‐serving biases.^51^, ^52^ By extension, and in accordance with a spectrum perspective on psychotomimetic compensation, a variety of psychotic‐like (e.g., conspiracy, apocalyptic or new‐age) beliefs might function as a schizotypal defense mechanism against uncertainty or social threat.^53^, ^54^

We realize that psychotic depression (PD) could be viewed as an exception to this proposed depression vs mania/psychosis dichotomy, and acknowledge that a lumping together of mania and psychosis may seem problematic, as oftentimes they do not occur together, and when they do cooccur, sometimes psychotic symptoms precede manic symptoms and sometimes manic symptoms precede psychosis. However, various features distinguish PD from psychotic disorders, such as intact insight,^55^, ^56^ less positive symptoms and less thought disorder.^57^, ^58^ These distinctions suggest that PD may represent, similar to the prodrome, remission or chronic phase of schizophrenia, a blunted form of psychosis—characterized predominantly by negative symptoms^58^—which in turn may correspond to blunted dissociative or dopaminergic drive (see Figure 3), or in some cases an antecedent phase of illness.

Indeed, individuals with PD are at high risk of converting to schizophrenia and bipolar disorder.^59^, ^60^ Psychosis is also generally more common and severe in mania than depression, and signs of mania such as increased activity and reduced sleep are common during acute psychotic episodes.^61^ Regarding the phenomenological relationship between chronic schizophrenia to bipolarity; frequent mood shifts and quick transitions between negative symptoms (associated with depression) and positive symptoms (associated with mania)^58^ can be observed in some cases of incipient and chronic psychosis.^62^

**FIGURE 2:**
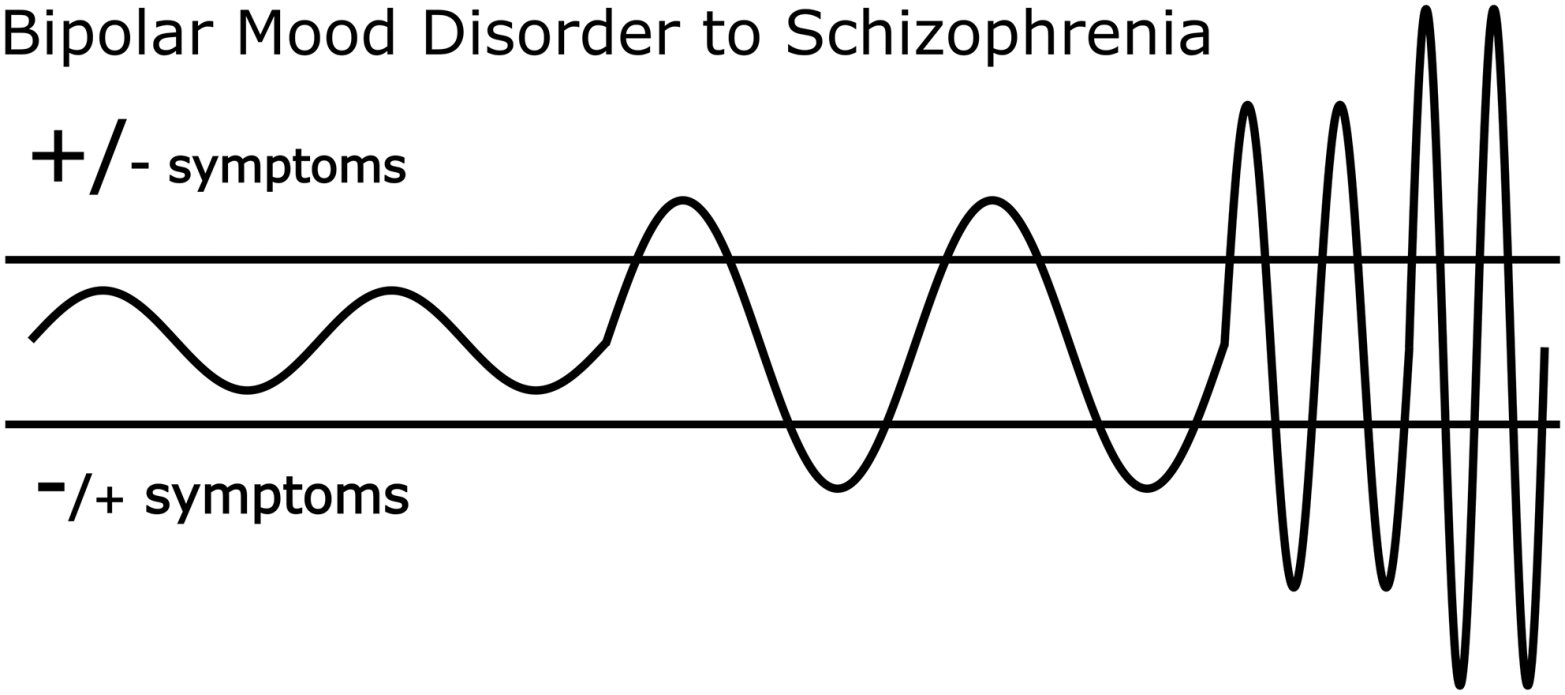
A visual representation of how longer duration affective shifts without psychotic features could gradually turn into shorter duration affective shifts with psychotic features at the poles, and finally into apparently “nonaffective” rapid psychotic shifts between negative and positive symptoms. In line with our model, it is also possible that psychotomimetic compensation drives affect switching, and that above a certain manic threshold (top bar) positive psychotic symptoms are more likely, while below a certain depression threshold (bottom bar) negative symptoms are more likely.

Studies have found that variation in positive beliefs about self predict paranoia,^63^ and that rapid symptom fluctuations predict violence, mediated by anger,^64^ in the context of first episode psychosis. John Custance,^65^ pseudonymous author of *Wisdom, Folly and Madness*, also perceived a similarity in the longer‐duration affective and cognitive shifts in individuals diagnosed with bipolar disorder and similar but more frequent shifts seen in some individuals diagnosed with schizophrenia; shifts which may occur on an hourly or even second by second basis (see also^66^). Indeed, our psychotomimetic sensitization model suggests that over time psychotic experiences can become more frequent and severe (see Figure 2).

Quick transitions from negative (tired/distracted) to positive (emotional/visionary) states on the order of seconds to minutes, are also characteristic of the temporal structure of naturally occurring religious/spiritual experiences.^67^, ^68^ And although illness, despair and grief do often precede these transitions^69^, ^70^—it would be misleading to interpret religious/spiritual experiences—which are often rare, unique, life events—as signs of mental disorder (although recurring spiritual/religious experiences are also quite common^68^). Moreover, while we focus here on psychotomimetic compensation as a response to stress—it should be noted that psychotic‐like experiences, or altered states of consciousness (ASCs), also occur during transitions between sleep and wakefulness, and while there are interesting interactions to explore between stress responses and fluctuations in arousal^71^, ^72^—these questions fall beyond the purview of this piece.

## 4 PSYCHOPHARMACOLOGY OF PSYCHOTOMIMETIC COMPENSATION

**FIGURE 3:**
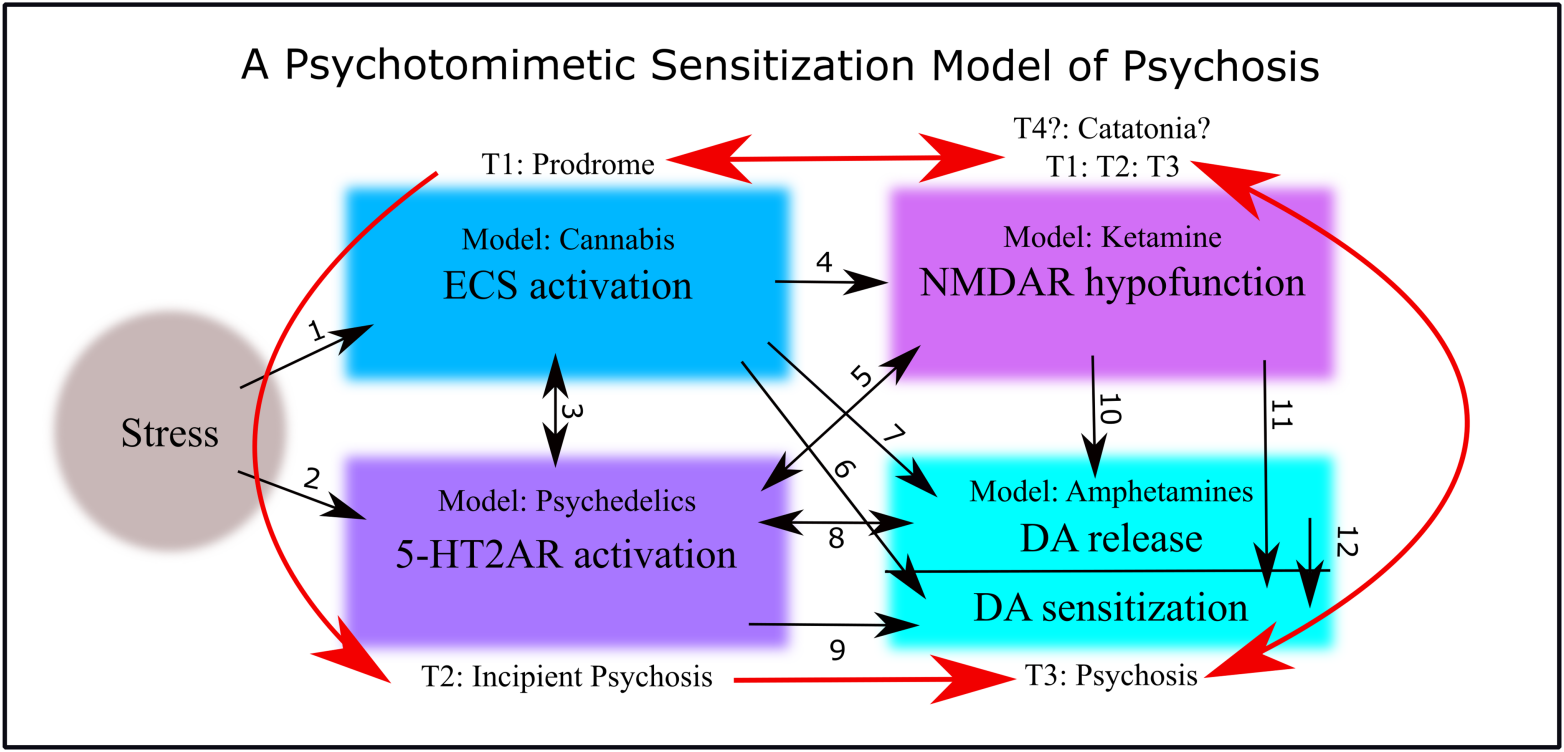
Intense stress engages the ECS, upregulates and activates serotonin 2A receptors (5‐HT2ARs), downregulates NMDA receptor activity, and induces transient dopamine (DA) release and subsequent DA sensitization. Normally this process is adaptive and promotes transient neuroprotection, neuroplasticity and behavioral activation in response to stressors, followed by a subsequent return to baseline. Reciprocal cross‐sensitization and positive feedback between these systems of over time, however, via intermittent exposure to stress or drugs, and perhaps dysregulated basal neurotransmission, can contribute to psychotic disorder (red arrows). Time points T1, T2, and T3 represent prodrome, incipient psychosis and psychosis, and are listed next to a good drug model of that particular stage(s). Each system is always interacting, and the numbered black arrows indicate positive effects of one system on another. References for each numbered arrow are listed as follows. Reviews replace lists of experimental studies when possible (noted in parentheses) 1: Stress—ECS (review)^73^ 2: Stress ‐5‐HT2AR (review)^82^, ^98^ 3: ECS—5‐HT2AR (review)^99^ 4: ECS—NMDAR hypofunction (review)^100^ 5: 5‐HT2AR—NMDAR hypofunction (no review, experimental study list)^101^, ^102^, ^103^, ^104^, ^105^, ^106^, ^107^, ^108^, ^109^, ^110^, ^111^, ^112^, ^113^, ^114^, ^115^ 6: ECS—DA release (review)^116^, ^117^, ^118^; also see^119^ for recent study 7: ECS—DA sensitization (no review, experimental study list)^120^, ^121^, ^122^, ^123^, ^124^, ^125^, ^126^, ^127^, ^128^, ^129^, ^130^, ^131^, ^132^; although see^133^, ^134^ 8: 5‐HT2AR—DA release (review)^135^ 9: 5‐HT2AR—DA sensitization (review)^135^ 10: NMDAR hypofunction—DA release (review and recent study)^106^, ^136^ 11: NMDAR hypofunction—DA sensitization (no review, experimental study list)^106^, ^137^, ^138^ 12: DA release—DA sensitization (review and recent study).^139^, ^140^

Endogenous psychotomimetic compensation is mediated by the same neurotransmitter/modulator systems engaged by psychotomimetic drugs such as cannabis, psychedelics, dissociative anesthetics, and stimulants (Figure 3). The endocannabinoid system (ECS), for example, modulates responses to homeostatic changes and various stressors,^73^, ^74^ and mediates adaptations to social defeat.^75^, ^76^, ^77^, ^78^, ^79^ The ECS is also believed to be responsible for the euphoric runner's high that occurs during high intensity endurance exercise.^80^, ^81^

Various stressors also upregulate, sensitize, and activate 5‐HT2A receptors which can have hyperthermic, anti‐inflammatory, antinociceptive and adaptive learning effects (as reviewed in Ref. [82]; also see [83]). Preclinical trials show that partial antagonism of NMDA receptors can protect against ischemia,^84^ hypoglycemia,^85^ seizure,^86^ traumatic brain injury,^87^ or drug‐related toxicity^88^ but timing matters. To be neuroprotective, *N*‐methyl‐d‐aspartate Receptor (NMDAR) antagonism must occur within a therapeutic time window surrounding the physiological insult.^33^, ^89^, ^90^

Common acute effects of cannabis, psychedelics, and disassociatives, as well as acute stressors such as exercise, fasting, hyperthermia and psychosocial stress, include a transient increase in brain‐derived neurotrophic factor (BDNF) which is implicated in neuroprotection and neuroplasticity.^91^, ^92^, ^93^, ^94^, ^95^, ^96^, ^97^ All psychotomimetics and various acute stressors also (typically) increase phasic dopamine release and subsequent DA sensitization, which is crucial to understanding how individuals are cross‐sensitized to psychosis via stress and/or drugs (see Figure 3; and below).

It is beyond the purview of this review to examine, in detail, all the compensatory functions and interactions between relevant neurotransmitter systems (Figure 3). So instead, we will focus on the compensatory potential of one exogenous psychotomimetic agent, classic serotonin (5‐HT) 2A receptor (5‐HT2AR) agonist psychedelics, and one endogenous psychotomimetic agent, kynurenic acid (KYNA).

## 5 CLASSIC PSYCHEDELIC COMPENSATION: 5‐HT2AR ACTIVATION

There are clear parallels between the temporal trajectory of the psychedelic experience and the trajectory of endogenous psychotomimetic compensation. Recall that a period of discomfort often precedes a mild dissociative, hypomanic or psychotic‐like “high” (e.g., runners high) in cases of intense physical stress (also see Figure 1). Similarly, a moderate or high dose of psilocybin containing mushrooms often first elicits feelings of nervousness, anxiety, confusion, nausea, being uncomfortable or lethargic.^141^ After this onset or “comeup” phase, a peak psychedelic experience occurs, followed by what is often described as pleasurable “comedown” and “afterglow”.^141^, ^142^ Interestingly, the phenomenology of the psychedelic mushroom comeup maps reasonably well onto the illness‐like symptoms reported during fever, hypoglycemia, ketoacidosis, and panic attacks which include nausea, dizziness, confusion, rapid or deep breathing and anxiety.^143^, ^144^, ^145^ These symptoms are also evident in acute stress reactions and disorders caused by traumatic events.^146^

One explanation for these phenomenological similarities is that stress‐induced 5‐HT2AR activation functions, in part, to increase access to energy (e.g., stored glucose) and increase ATP synthesis and metabolism^31^, ^147^, ^148^, ^149^ in demanding circumstances, as well as to help buffer the organism against the excitotoxic effects of excessive energy demands or compromised metabolic function.^31^, ^150^, ^151^

Psychedelic activation of 5‐HT2ARs promotes mitochondrial biogenesis,^30^, ^152^, ^153^ resulting in increased expression of antioxidant enzymes, decreased cellular reactive oxygen species, and enhanced neuroprotection against excitotoxic and oxidative stress^30^ (also see Ref. [31] for review). Consistent with these effects, the psychedelic agent N,N‐dimethyltryptamine (DMT) reduces infarct size and improves functional recovery following experimentally‐induced cerebral ischemia in rodents and psychedelics more generally are under development for stroke, as well as neurodegenerative conditions such as Alzheimer's disease.^154^

In regard to how metabolic stress or energy deficits trigger a psychedelic‐like serotonergic response, it has been proposed that chemosensitive serotonin neurons in the brainstem are responsive to changes in pH,^155^, ^156^ which is an expected consequence of intense exercise,^157^ hypoxia,^158^ inflammation,^159^ ketosis^160^ and other states linked to psychotomimetic compensation such as near‐death experiences (NDEs). Some scholars point to endogenous psychedelic release during NDEs,^161^ which is also consistent with hypoxia‐induced rerouting of tryptophan metabolism towards increased tryptamine production,^162^ and the fact that NDEs occurring due to cardiac arrest are associated with hypoxia and increased end‐tidal carbon dioxide (CO_2_) levels.^163^

There is some debate as to the similarity of NDEs and psychedelic experiences,^164^, ^165^ but the visionary and emotional characteristics and effects of these experiences are quite similar^166^, ^167^, ^168^ as are their temporal trajectories. A substantial subset of distressing NDEs are characterized at first by panic and fear, but subsequently turn into positive experiences of calm, peace and love.^169^ A similar phenomenological transition from panic to peace is described in experiences of drowning.^170^ It is noteworthy in this regard that the 5‐HT2AR mediates CO_2_ induced arousal,^171^ and that CO_2_ inhalation elicits feelings of anxiety and panic^172^, ^173^ as well as (less‐well‐known) cathartic, pleasurable and visionary experiences.^174^, ^175^ One of the possible negative consequences of psychedelic use is also an increase in anxiety and panic.^176^, ^177^

Psychotomimetic responses to respiratory and metabolic stress map onto the symptoms and phenomenological trajectories of psychotic disorders as well. Panic attacks elicit mental imagery, strong emotions and a sensed loss of control^144^—subjective features that relate to psychedelic and incipient psychotic experiences. Indeed, anxiety and panic often precede first‐episode psychosis,^178^, ^179^ and panic symptoms are common and severe in psychotic disorders.^178^ Additionally, both psychosis and mania are linked to low brain pH^180^, ^181^, ^182^ (although see Ref. [183])—which, as mentioned above—may be a common trigger of psychotomimetic compensation, whether elicited by changes in respiration or metabolism. The putative links between respiratory and metabolic crises and psychotomimetic compensation could help explain some of the paradoxical links between respiratory, metabolic and psychotic disorders; for example, how it is that respiratory and metabolic disorders increase the risk of psychosis, while treating psychosis with antipsychotics further increases risk of respiratory and metabolic disorders.^184^, ^185^

Christopher Palmer^151^ convincingly argues that psychiatric disorders including schizophrenia and bipolar disorder are caused in part by compromised metabolic function, and it is possible that psychotic and manic symptoms result, in part, from an attempt to compensate against metabolic stress.^186^ Although in need of replication, an older study by Kitay and Altschule^187^ found that 40% of individuals hospitalized with psychosis had ketone levels—a primary alternative fuel sources for humans –above the upper limit seen in controls. Other studies have found increased lactate levels—another potent alternative fuel source^188^—in schizophrenia and bipolar disorders.^180^, ^189^

Although atypical antipsychotics (5‐HT2AR blockers) and mood stabilizers help treat the symptoms of psychosis and mania, they often contribute to metabolic dysfunction and related health issues such as diabetes and obesity.^185^, ^190^ Conversely, preliminary data suggests ketogenic diets may be useful in treating psychiatric disorders.^191^, ^192^, ^193^ One explanation for the putative antipsychotic and antiepileptic effects of ketogenic diet is that, by providing access to a viable alternative fuel source, or by promoting stable increases in neuroprotective KYNA,^194^, ^195^, ^196^, ^197^, ^198^ the ketogenic diet dampens phasic psychotomimetic compensation mediated, for example, by activation of 5‐HT2ARs. It follows theoretically that it may be the bipolar fluctuations between depressive and manic symptoms, or negative and positive symptoms, or between abstinence and binge drug use, that contributes to bipolar, psychotic and substance abuse disorders.

Having touched upon psychotomimetic compensations to physiological stress, let us now turn towards the socioemotional trajectory of psychotomimetic compensation—as modeled by psychedelics. Psychedelics often elicit and then resolve stressful or challenging experiences, a process that is associated with long‐term increases in well‐being.^199^ Despite a dearth of research on the temporal trajectory of the psychedelic experience,^141^ descriptions of emotional breakthroughs,^199^ catharsis, revised life priorities,^200^ renewed senses of purpose, heroic doses and journeys, and death‐rebirth experiences^201^ all suggest that these experiences have the temporal structure of a “problem(T1)‐solving(T2) experience” that has been related to spiritual and acute psychotic experiences in the past.^202^

The transition from an uncomfortable psychedelic comeup to a relieving comedown also maps reasonably well onto relevant clinical transitions from depression to mania, and from psychotic prodrome to first‐episode psychosis.^67^ In subjective reports of transitions from depression/prodrome towards mania/psychosis individuals often first report feeling better, temporarily, but then experience increased distress associated with enduring hyperactive or psychotic experiences.^47^, ^203^ We notice a similar distress when psychedelic mushroom experiences endure longer than hoped for, leading to exhaustion, further panic, or the inability to sleep or return to a comfortable baseline.^141^

One risk associated with the antidepressant use of psychotomimetic drugs, sleep deprivation, fasting or ketogenic diet is the possibility of triggering a switch towards mania and psychosis.^204^, ^205^, ^206^ There are plenty of examples, however, when compensatory switches—even ones that resemble mania or psychosis—do not lead to progressive dysregulation and dysfunction. The transition from initial shamanic sickness to shamanic death‐rebirth experience and shamanic election^207^ is one example, as are innumerable other types of salvific, spiritual or secular self‐transformative experiences.^68^, ^82^

## 6 PSYCHOTOMIMETIC INTERACTIONS

The fact that we observe a switch from an aversive illness‐like state to a state of relief in the psychedelic mushroom experience^141^ suggests that interactions and downstream contributions from non‐serotonergic NT systems influence the trajectory of psychedelic experiences (see Figure 3 for references and reviews). Building on the ideas of Freedman,^208^ Marona‐Lewicka and colleagues^209^ suggest that there are two distinct temporal phases to the LSD experience, the first psychedelic and mediated by 5‐HT2AR activation and the second manic/paranoid and mediated by dopamine (DA) signaling.

Although LSD has more affinity for dopamine receptors than some other psychedelics like psilocybin, Vollenweider and colleagues,^8^ found that DA subtype 2 receptor blockade reduced oceanic boundlessness, increased dread of ego‐dissolution, and had no effect on visionary restructuralization in a group of healthy volunteers given psilocybin.^8^ In another study haloperidol (D2R antagonist) pretreatment led to increased death in rodents given LSD.^210^ Thus, it appears that dopamine signaling may play an important role in all psychedelic experiences, perhaps particularly in the transition from uncomfortable comeup (associated with rising 5‐HT2AR activation) towards peak experience, emotional breakthrough and pleasurable come‐down.

Similarly, regional alterations in glutamate are implicated in both negative and positive experiences of ego‐dissolution under psilocybin.^211^ It has recently been shown that concurrent use of lithium—with putative effects on DA and NMDAR neurotransmission—drastically increases risk of seizures and bad trips on psychedelics.^212^ Taken together, this all suggests that the positive effects of psychedelic 5‐HT2AR agonism are somewhat dependent upon interactions with other NT systems. Moreover, it is possible, perhaps even likely, that the psychotomimetic engagement of other NT systems (see Figure 2) exert counterbalancing effects on 5‐HT2AR agonism. For example, psychedelics increase body temperature,^213^ while cannabinoid receptor agonists,^214^ D2R agonists^215^ and NMDAR antagonists all reduce body temperature.^216^

The complex interplay of NT systems involved in psychotomimetic compensation (Figure 2), as alluded to by the limited number examples provided above, gives us a preliminary sense of why psychotic‐like experiences occur in contexts of stress and energy deficits, and why, although antipsychotics can help treat symptoms of psychosis, they can also contribute to weight gain,^217^ metabolic disorder,^218^, ^219^ susceptibility to heat stress^220^ and related life‐threatening conditions such as neuroleptic malignant syndrome.^221^

## 7 KYNURENIC ACID: ENDOGENOUS NMDAR ANTAGONISM

Elevated levels of glutamate can lead to excitotoxicity via NMDA receptor activation, Ca^2+^ influx, oxidative stress and mitochondrial dysfunction, a process which may be protected against, to some extent, by a compensatory increase in brain kynurenic acid (KYNA); an NMDA receptor antagonist.^222^ Animal models also show that KYNA increases energy expenditure and improves metabolic function.^223^

In line with our psychotomimetic compensation model, increased neurotoxic quinolinic acid (an NMDA receptor agonist) and decreased KYNA is seen in depression,^224^, ^225^ while increased levels of KYNA is associated with mania and psychosis^226^, ^227^, ^228^, ^229^, ^230^ (although see Ref. [231, 232]). Stress‐induced increases in salivary KYNA are elevated in individuals diagnosed with schizophrenia,^233^, ^234^ and increased levels of KYNA are associated with deficits in pre‐pulse inhibiton and audio‐sensory gating in animal models of schizophrenia (see Ref. [228] for review).

The belief that endogenous antagonism of NMDARs plays a role in the pathophysiology of schizophrenia^235^ is supported by the fact that exogenous NMDAR antagonism, via ketamine for example, is a good pharmacological model of the negative and positive symptoms of psychosis.^236^ Ketamine, however, is also a rapid acting antidepressant.^13^ Other conventional antidepressants,^237^ and non‐pharmacological interventions for depression such as exercise,^238^ sleep deprivation^239^, ^240^ and fasting^241^ also increase levels of KYNA. Although not known at the time of writing, a clear prediction is that, like ketamine,^242^ classic psychedelics (e.g., LSD, psilocybin) will also increase central nervous system levels of neuroprotective kynurenic acid (KYNA).

## 8 PSYCHOTOMIMETIC SENSITIZATION

Functional psychotic disorders like schizophrenia are increasingly conceived of as neurodevelopmental disorders, but accumulation of life stress or drug abuse is still very much implicated in their development.^243^ Figure 3 (see above) presents a unified (albeit simplified) model of how predisposed individuals can be sensitized to psychosis over‐time via recurrent stress, recurrent drug use or both, all via shared pathways. From a developmental perspective the idea of psychotomimetic sensitization fits observations that first episodes of psychosis develop much more slowly than recurrent episodes.^244^ The initial schizophrenia prodrome can last years, and manic psychoses often take years to manifest after the onset of depressive symptoms.^59^ In contrast, subsequent psychotic episodes emerge swiftly and with few warning signs.^244^

Psychotic disorders are highly heritable, but this fact does not contradict the model presented here, which highlights the etiological role played by stress and psychotomimetic drug exposure. Developmental vulnerability to psychosis in the absence of repeated drug use suggests an endogenous psychotomimetic process that is most likely facilitated by chronic or high‐dose stress during critical developmental windows.^82^, ^243^ Prenatal and early life stress predisposes individuals to psychosis, and individuals with schizophrenia show increased dopamine release in response to stress, an effect which is mediated by the 5‐HT2AR ^245^, ^246^, ^247^ and primed via activation of the ECS.^129^, ^130^ Dopaminergic sensitization develops in response to and then eventually reinforces psychotomimetic signaling, producing a self‐perpetuating feedback loop.

A single administration of a dopamine‐enhancing drug does not typically induce the psychotic‐like experiences that are elicited by other psychotomimetics.^248^, ^249^ Chronic and high doses of dopamine‐enhancing drugs do induce psychotic‐like phenomena, alter NMDA receptor functioning and expression,^248^, ^249^, ^250^ and increase 5‐HT2AR sensitivity and activity.^251^, ^252^, ^253^ Antagonizing 5‐HT2ARs in turn has antipsychotic effects in patients with schizophrenia and those receiving L‐DOPA for Parkinson's disease.^254^, ^255^ These synergisms begin to reveal how psychotic experiences emerge, are reinforced, and sensitized over time via the same processes implicated in learning and addiction and that are implicated in the psychotomimetic effects of psychedelics. What at first can be a benign or positive response to stress or drugs, can over‐time begin to resemble a dysfunctional disorder that is beyond the control of the individual.

Acknowledging that a susceptibility to psychosis develops over time; psychosis itself is much more likely to manifest when—borrowing terminology from psychedelics—a person's set (mindset) and setting (social context) are negative.^256^ Indeed, negative mindsets are associated with mental phenomena seen in psychosis, including jumping to conclusions,^257^ delusions and hallucinations.^258^ In regard to social context, a threat to or loss of social status is also believed to make people more vulnerable schizophrenia.^20^, ^259^, ^260^, ^261^, ^262^ The same “set and setting” context‐dependency holds true for drug‐induced psychotic‐like experiences.

Experiences of childhood trauma increase psychotic symptoms elicited by amphetamines,^263^ neuroticism predicts challenging experiences with psychedelics^264^ and psychedelic use in emotionally alienating, non‐supportive environments increases the risk of problematic psychotic‐like (often paranoid) reactions. Thus, while we have been discussing sensitization to psychotomimetic compensation in the broader sense, there is a case to be made that specific symptoms of psychosis—e.g., derogatory auditory verbal hallucinations—reflect a sensitization to specific stimuli (e.g., social cues) and emotions (e.g., shame).^29^ Nevertheless, there are also noticeable trends that support a broader conception of psychotomimetic sensitization, such as a general convergence towards paranoia in emerging psychotic disorders, or the increased preponderance of auditory hallucinations and delusions with extended time awake in sleep deprivation models of psychosis.^26^

Therefore, while we agree with the consensus that psychedelic experiences tend to be positive when set and setting are positive, we note that a process of psychotomimetic sensitization could still increase susceptibility to psychotic‐like experiences in negative future contexts; with younger and more frequent poly‐substance users being at greater risk.

## 9 DO PSYCHEDELICS INCREASE THE RISK OF PSYCHOSIS?

It is beyond the purview of this piece to address risk factors associated with each commonly used psychotomimetic agent. However, links between cannabis use and psychosis have been firmly established.^9^, ^265^, ^266^ It is also generally accepted that chronic high‐dose methamphetamine use increases the risk of psychosis.^267^ Less attention has been directed at the risks associated with the use of subanesthetic doses of dissociative anesthetics such as ketamine and use of classic psychedelics.

A longitudinal study of 2588 adolescents in Munich, Germany found that use of psychedelics was positively associated with lifetime experience of psychotic symptoms.^268^ More recently, two large‐scale, population‐based studies report that prior year use of LSD predicts increased depression, suicidal ideation, and presence of any/serious mental health condition.^268^, ^269^ Conversely, Krebs and Johansen^270^ report that lifetime use of psychedelics is not an independent risk factor for mental health problems or psychotic symptoms when other risk factors are taken into account. However, the unadjusted data in Krebs and Johansen^270^ shows double the rates of psychotic symptoms among people who have ever used psychedelics (see fig. 3 of Ref. [270, 271]; 50). Survey data available to the public also show that individuals in the USA who have ever used hallucinogens (which according SAMSHA operationalization includes classic psychedelics, ecstasy/MDMA and dissociatives) are about twice as likely to have received inpatient mental health treatment within the last year prior to survey (data visualization available at https://pdas.samhsa.gov/#/).

Of course, this increased risk could be due to other confounding variables associated with psychedelic use, such as risk‐taking, or other substance use, and there is some debate over whether the use of other controlled substances, including cannabis, should be controlled for in analyses of risks associated with psychedelics.^271^, ^272^ According to data from Krebs and Johansen,^270^ 98% percent of people who had ever used psychedelics also tried cannabis. Although it has been argued that drawing conclusions from the very niche population that only uses psychedelics has limited generalizability, data from such a study might provide information essential for estimating the actual effect size of the direct risk from psychedelic use. However, given the known influences of set and setting on the psychedelic experience, and our proposed psychotomimetic sensitization model, which highlights relevant interactions between psychotomimetic drug effects, it may not always be appropriate to isolate the effects of psychedelics from contextual confounds, or to only report/highlight adjusted odds ratios. At the very least, caution needs to be taken when communicating information to the public. As Johnstad^272^ suggests, widespread adoption of psychedelics on a society‐wide level could promote a scenario in which occasional psychedelic use increases quality of life in already well‐adjusted individuals and promotes dysfunction when used frequently or in more negative contexts.

Controlling for mental illness among users of psychedelics is also a debatable analytic strategy, particularly if we entertain the possibility that psychedelic use could increase susceptibility to psychosis, or that other mental health factors could influence the effects of psychedelic use. The adage that psychedelics only cause psychosis in vulnerable individuals avoids the issue, considering that the number of vulnerable individuals is far greater than the number of people who go on to develop a psychotic disorder.

Within the USA the most common ages to first use hallucinogens (16–18) and cannabis (15–18) precede the typical age of onset of schizophrenia‐spectrum disorders,^273^ supporting the possibility of a causal effect (data visualization available at https://pdas.samhsa.gov/#/). The number of people reporting psychotic or other adverse reactions to psychedelics is admittedly low,^274^ but may be underreported, and different methods of collecting data (e.g., internet survey, home interviews, patient data) might generate pictures of relative risk different from those obtained from carefully controlled and psychotherapeutically‐supported clinical trials. Interviews of individuals referred to early intervention services for first‐episode psychosis, for example, reveal rates of hallucinogen use that are much higher than that observed in the general population.^275^, ^276^

A recent meta‐analysis suggests that the transition to schizophrenia occurs in 26% of hallucinogen‐induced psychoses, compared to 34% and 22% of psychoses induced by cannabis and amphetamines, respectively.^10^ These transition rates are comparable to transition rates from brief and atypical psychoses, and psychoses not otherwise specified (36%), but are higher than transition rates from other types of drug‐induced psychosis (e.g., from alcohol or opioids).^10^ However, other variables, such as age, may account for some of these differences.^10^ Kendler et al.^277^ found that the likelihood of conversion to schizophrenia does decrease with the age of first substance‐induced psychosis, and that the mean time to schizophrenia conversion after substance‐induced psychosis is 39 months. These observations roughly conform to Boutros et al.^278^ observations that peak substance related admissions to Connecticut (USA) state hospitals during the late 1960s and early 1970s preceded a sharp rise in psychotic and mood disorder diagnoses some five years later.

In summary, current evidence suggests that we should not prematurely dismiss the psychotogenic risks posed by psychedelics, particularly within populations most likely to use these agents outside of clinical contexts, both now and in all possible futures. The possibility of psychotomimetic compensation and sensitization provide additional reasons for being cautious when trying to assess independent risks associated with psychedelic use. Models of risk must be grounded in theory, and there are theoretical reasons to suspect that adverse life events, other substance use, and mental health factors will interact with psychedelic use in determining the risk of psychosis or other mental health outcomes.

### 9.1 Nomenclature of targets and ligands

Key protein targets and ligands in this article are hyperlinked to corresponding entries in http://www.guidetopharmacology.org, the common portal for data from the IUPHAR/BPS Guide to PHARMACOLOGY,^284^ and are permanently archived in the Concise Guide to PHARMACOLOGY 2019/20.^285^

## 10 CONCLUSIONS, IMPLICATIONS, AND LIMITATIONS

Psychedelics elicit positive psychological and spiritual experiences as well as experiences that resemble psychosis. We explain this apparent paradox by appealing to the process of psychotomimetic compensation, which is generally therapeutic at short durations and infrequent intervals but can become pathogenic when chronic or frequent via a process of psychotomimetic sensitization. This model is generally consistent with the hormetic effects of short‐duration stressors^279^ and the idea of canalization—the entrenchment and stabilization of thought and behavior—as applied to psychopathology.^280^ Use of psychedelics is associated with an increased risk of psychotic symptom development in the general population, although any attempt to isolate the risk ascribable to psychedelics alone poses theoretical problems. Individuals prone to psychosis are also more likely to use psychotomimetics,^281^, ^282^ questioning the directionality of any association, although a compensatory use of psychotomimetics, as a form of coping, would be consistent with our framework. A few practical implications follow. First, we see no reason to prematurely dismiss a psychotomimetic perspective on psychedelics. This perspective, if approached in a balanced way, might help us understand and mitigate the risks associated with the therapeutic and recreational use of psychedelics, as well as to understand psychosis.

There are also limitations to our analysis, and potential risks associated with pathologizing and suppressing compensatory psychotomimetic processes. By highlighting the importance of the duration, frequency, and intensity of psychotomimetic experiences, we limit consideration of other context‐specific factors that influence the form and outcome of these experiences, such as controllability, aesthetics and social environment. In some cases, controlling the contexts in which psychotomimetic experiences occur will yield more benefits than trying to suppress the emergence of these experiences altogether, which could unintentionally favor the emergence of psychotic symptoms during periods of inattention, reduced control and decreased social support. Shamanic cures to psychotic‐like developmental crises are found all over the world in forager cultures with few class or political distinctions, and are achieved by gaining a mastery over (perceived) spirits that can otherwise persecute and destroy the individual.^207^ Shamanic rituals share many parallels with common and ideal “settings” for recreational and therapeutic use of psychedelics.^201^ Aesthetic manipulations (eyeshades, music, visualization techniques) favor the emergence of visual and multimodal as opposed to auditory hallucinations, and repetitive movements (dancing) and collective rituals contribute to feelings of positive emotion and control. Social environment and preparation might likewise be leveraged to promote prosocial as opposed to antisocial attitudes and beliefs, although addressing the relationship between values and mental health raises complex ethical and methodological hurdles.

Our presentation of psychotomimetic sensitization is further limited in two important ways. First, we do not address the importance of periodic motivational and attentional deficits in promoting the psychotomimetic sensitization process, which is consistent with how sensitization to drugs occurs, via periodic abstinence and binges.^139^ Second, mechanisms unrelated or only peripherally related to sensitization also contribute to psychotic disorder. An alternative explanation to our sensitization model would be that increased psychotomimetic compensation over time simply reflects increased brain or metabolic dysfunction^14^, ^151^—although we cannot discount the possibility that such dysfunction would contribute to a process of sensitization—in fact we expect that it would. These considerations, along with those mentioned in the previous paragraph do not support the idea of a clear linear relationship between number of psychotomimetic experiences and pathology. It remains to be seen whether controlled engagement with psychotomimetic experiences, for example via psychospiritual practices or the therapeutically‐constrained use of psychedelics, could reduce symptoms of psychosis or the distress associated with these symptoms.^283^

In conclusion, psychotomimetic experiences are relatively normal, fall on a spectrum, and function as compensations against stressors of all sorts. It follows that they are neither abnormal nor miraculous experiences, and should not be categorially suppressed or promoted, but rather engaged with more neutrally in accordance with the specific needs of individuals and communities. The suitability of personal solutions to environmental challenges cannot and should not be reduced to simple, context‐independent, biocentric explanations.

## AUTHOR CONTRIBUTIONS

AB and CLR wrote the manuscript. AB, CLR, and RCH revised the manuscript.

## FUNDING INFORMATION

Funding was provided to CLR via the Mary Sue and Mike Shannon Distinguished Chair for Healthy Minds, Children and Families and to AB via an academic scholarship from Usona Institute. The funders had no role in study design; in the collection, analysis, and interpretation of data; in the writing of the report; or in the decision to submit the article for publication.

## ETHICS STATEMENT

Ethics approval was not required for this manuscript.

## Data Availability

No new data were created or analysed in this study. Data sharing is not applicable to this article.
